# Congenital Transmission by Protozoan

**DOI:** 10.1155/2012/173437

**Published:** 2012-10-17

**Authors:** Ricardo E. Fretes, Ulrike Kemmerling, Demba Sarr

**Affiliations:** ^1^Cell Biology, Histology, and Embryology Department, Medicine School, Universidad Nacional de Córdoba, 5000 Córdoba, Argentina; ^2^Universidad Nacional de La Rioja, 5300 La Rioja, Argentina; ^3^Program of Anatomy and Developmental Biology, Institute for Biomedical Sciences, Faculty of Medicine, University of Chile, 8380453 Santiago, Chile; ^4^Department of Infectious Diseases and Center for Tropical and Emerging Global Diseases, University of Georgia, Athens, GA 30602, USA

Congenital transmission by protozoan parasites is a worldwide important public health problem. Congenital infections affects the mother and the fetus or newborn. It is still surprising that despite the abundant immunoepidemiological knowledge of congenital transmission of protozoan parasite, no definite etiology or predictive diagnostic tests have been identified. Understanding the mechanisms by which host/parasites infection and interaction occurs is one of the most important topics that will help find specific biomarkers of infection, prevent congenital transmission, maintain the health of the newborn, and develop safe and efficient treatments. In addition, understanding of the biological mechanisms of host/parasite interactions will facilitate protection of mothers and their families and reduce costs in health services. Moreover, this will lead to gain insight into epidemiological aspects and association with other pathologies and preserve the wellbeing of the newborn. In this special issue we have invited a few papers that address such issues.

Congenital malaria is underestimated and usually associated with low-density cord parasitemia. However, it is increasingly recognized as a potentially serious complication of maternal malaria during pregnancy in Sub-Saharian Africa. Earliest epidemiological studies have reported prevalence varying widely in malaria-endemic areas from 0% to 33%. At birth, infections are usually asymptomatic with low parasitemia and the diagnosis by microscopy is often missed. Infection may occur by transplacental passage of parasites during disruption of the placental barrier at the time of delivery, with subsequent clinical illness in the newborn baby. A paper of this issue assessed the prevalence of congenital malaria during the dry season (period of low-mosquito density and low-malaria incidence) using peripheral and cord blood smears of new born babies in association with peripheral and placental blood smears of, respectively, near-term and term pregnant women. This study is interesting because the authors found that congenital malaria is not rare in their study area. Another paper is a literature review on the prevalence, burden, diagnosis, prevention, and control of congenital malaria in Sub-Saharian Africa. This review is of interest to individuals working in clinics and laboratory diagnostic of malaria but also to national governments and partners in Sub-Saharian Africa. The authors highlight the challenges in parasitological and clinical diagnosis, the challenges in prevention with the use of intermittent preventive treatment (IPT) and insecticide-treated nets (ITNs) and provides recommendation on how to strengthen the health system in Africa. Another paper reports the prevalence of transplacental malaria in Burkina Faso, a West African country, and determines the real burden of transplacental transmission. The authors show associations between levels of parasite in the maternal, placental, and umbilical cord blood. All papers about malaria published in this special issue show the interest of pursuing investigations for a better understanding of vertical transmission by malarial parasites.

During congenital Chagas transmission, the parasite reaches the fetus by crossing the placental barrier. In the past few years congenital transmission of *T. cruzi* has increasingly become more important, and partly responsible for the “globalization of Chagas' disease,” constituting a public health problem of increasing relevance. The fact that only a percentage of the infected mothers transmit parasites to their fetuses raises the question of the ability of the placenta as well as the immunological status of mother and fetus/newborn to impair the parasite transmission. Therefore, it is thought that congenital Chagas disease is the product of a complex interaction between the parasite, the maternal, and fetus/newborn immune responses, and placental factors. Additionally, a paper of this special issue constitutes a morphological analysis by immunohistochemical and histochemical methods of placentas from women with chronic Chagas' disease. *T. cruzi*-infected placentas present destruction of the syncytiotrophoblast and villous stroma, selective disorganization of the basal lamina, and disorganization of collagen I in villous stroma. Changes in the extracellular matrix of placental tissues, together with the immunological status of mother and fetus, and parasite load may determine the probability of congenital transmission of *T. cruzi*. Another paper assayed the effect of *T. cruzi* on glucose transporter protein-1 (GLUT1), which is the main isoform involved in transplacental glucose transport. High glucose media as well as *T. cruzi* infection reduce GLUT1 expression. The effect of *T. cruzi* infection on GLUT1 expression may explain some of the clinical manifestation of congenital Chagas disease. Finally, a paper analyzing the congenital transmission of Chagas disease is a review about the role of possible local placental factors that contribute to the vertical transmission of the parasite. Additionally, in that review different available methods for studying the congenital transmission of *T. cruzi* are analyzed. In that context, the *ex vivo* infection of human placental chorionic villi by *T. cruzi *trypomastigotes constitutes an excellent tool for studying parasite infection strategies as well as possible local antiparasitic mechanisms.

*Ricardo E. Fretes* *Ricardo E. Fretes* *Ulrike Kemmerling* *Ulrike Kemmerling* *Demba Sarr* *Demba Sarr*

